# ATF-3/miR-590/GOLPH3 signaling pathway regulates proliferation of breast cancer

**DOI:** 10.1186/s12885-018-4031-4

**Published:** 2018-03-09

**Authors:** Qiong Song, Qiu Chen, Qimin Wang, Longqiu Yang, Dongdong Lv, Guangli Jin, Jiaying Liu, Baolin Li, Xuejie Fei

**Affiliations:** 1 Department of Anesthesiology, Zhengzhou Central Hospital Affiliated to Zhengzhou University, Number 195, Tongbai Road, Zhengzhou, Henan Province 450000 China; 2 Department of Hospital Infections, Shuguang Hospital Affiliated with Shanghai University of Traditional Chinese Medicine, Number 187, Puan Road, Shanghai, 200021 China

**Keywords:** Breast cancer, MiR-590-3p, ATF-3, GOLPH3, Proliferation, Cell cycle

## Abstract

**Background:**

Breast cancer is one of the leading causes of death in women worldwide. Fast growth is the important character of breast cancer, which makes sure the subsequent metastasize and invasion breast cancer. Golgi related genes GOLPH3 has been reported to regulate many kinds of cancers proliferation. However, its upregulator remains largely unknown. miRNA modulate gene expression by post-transcriptional repression to participate in many signaling pathway of breast cancer cell proliferation. miR-590 has been reported to regulate tumorgenesis and could be regulated by its own target ATF-3. But whether miR-590 can be the modulator of Golgi related genes to regulate the breast cancer proliferation is unclear.

**Methods:**

We performed the bioinformatics analysis of survival rate and expression differences of patients using the data of The Cancer Genome Atlas (TCGA).Both of MTS and BrdU assays were used for cell proliferation analysis. Cell cycle was detected by flow cytometry .qRT-PCR was used for detecting the cell cycle related gene expression. Student’s t-test or One way anova was used for statistics.

**Results:**

We found the upregulation of GOLPH3 in breast cancer samples compared with normal breast tissues, which also was related to the poor prognosis. Overexpression of GOLPH3 significantly promoted proliferation both of MDA-MB-231 cells (ER negative) and MCF-7 cells (ER positive). We further found that miRNA-590-3p could directly target the 3′-UTR of GOLPH3 mRNA to repress its expression. Overexpression of miR-590-3p inhibited the proliferation of MDA-MB-231 and MCF-7 cells. The rescue experiments indicated that overexpression of GOLPH3 significantly resorted the proliferation inhibited by miR-590-3p. We also found that ATF-3 repressed miR-590-3p expression to modulate miR-590/GOLPH3 pathway to regulate breast cancer cells proliferation.

**Conclusions:**

This study not only suggests that the ATF-3/miR-590/GOLPH3 signaling pathway is critically involved in the proliferation of breast cancer cells, but provides a novel therapeutic target and new insight base on epigenetic regulation for future breast cancer diagnosis and clinical treatment.

**Electronic supplementary material:**

The online version of this article (10.1186/s12885-018-4031-4) contains supplementary material, which is available to authorized users.

## Background

Breast cancer is one the most common cancers worldwide and results of the death among females [1]. Breast cancer cells have almost unlimited ability of survival with fast proliferation and invasion [2]. The Golgi apparatus, an organelle involved in post translational modification and sorting of proteins, is increasingly viewed as a platform for the spatial regulation of signaling molecules [3], and its function in directly regulating cancer genesis and development is widely accepted. The disassembly of Golgi was found in advanced androgen-refractory prostate cancer cells and primary prostate tumors and correlated with Gleason score and metastasis [4]. Depletion of YSK1 and MST4 alters Golgi structure and inhibits cell migration in Hela cells [5]. Golgi phosphoprotein 3 (GOLPH3) is a new oncogene that is closely related to the tumor growth, metastasis in some types of cancer. Upregulation of GOLPH also suggested the poor prognosis in epithelial ovarian cancer [6]. GOLPH3, a PI(4) P effector, enhances cell proliferation and tumorigenicity [7–9]. Overexpression of GOLPH3 is associated with poor clinical outcome in gastric cancer, non-small-cell lung cancer (NSCLC) and ovarian cancer [10–12]. In breast cancer, overexpression of GOLPH3 promotes proliferation and tumorigenicity [8, 13]. However, the mechanism of GOLPH3 participating in modulating proliferation and its upstream regulators in breast cancer should be researched deeply.

In addition to protein-coding genes, microRNAs (miRNAs) are endogenously expressed short noncoding RNAs. Through binding to 3′-untranslated region (3′-UTR), miRNAs can repress protein translation of the target mRNA to regulate many kinds of physiological process or disease incidence [14–16].Increasing number of studies suggested that miRNAs control genes involved in cellular processes such as inflammation, cell-cycle regulation, stress response, differentiation, apoptosis, and migration [17]. miR-181a expression was upregulated by TGF-beta signaling to promote breast cancer metastasis [18]. And miR-143/145 cluster is reported to inhibit tumor invasion in prostate cancer by targeting Golgi membrane protein 1 [19]. miR-126 can directly target the GOLPH3 to repress the proliferation in esophageal squamous cell carcinoma [20]. miR-590 has been reported to inhibit breast cancer cell stemness and metastasis by targeting SOX2 [21]. miR-590 also induced breast cancer cell apoptosis with the downregulation of JAK2, PI3K, MAPK1, and CREB that are related to the tumor genesis and development [22]. However, in breast cancer, whether miR-590 can modulate the Golgi associated proteins expression to regulate the breast cancer proliferation and its upstream regulator is unclear.

Activating transcription factor 3 (ATF3) is one of the members of the ATF/CREB transcription factor family and can be a stress-inducible protein responses to the signals include anoxia, carcinogens, DNA damage, radiation and so on [23].Basal expression of ATF3 is low in normal cells, while in several malignant cancer tissues the expression increases significantly [24, 25]. ATF3 can induce cells to enter the cell cycle from the stationary phase, thus accelerating cell proliferation, and this process is critical during the migration and invasion of tumor. ATF3 is reported to work as a regulator in myeloid cells that enhances breast cancer metastasis [26]. Previous study found that the expression of miR-590 was down regulated in human breast cancer and this could be regulated by ATF-3 [27]. But whether ATF-3 can regulate the Golgi associated genes signaling pathway during the breast cancer genesis and development remain largely unknown.

Our research indicated that overexpression of miR-590-3p could inhibit the proliferation of MDA-MB-231 and MCF-7 cells. While miRNA-590-3p could directly target the 3′-UTR of GOLPH3 mRNA and repressed its expression. GOLPH3 mediated the function of miR-590-3p and formed the miR-590/GOLPH3 signaling pathway. By repressing miR-590-3p, ATF-3 modulated the miR-590/GOLPH3 signaling pathway on regulating proliferation of breast cancer cells. This study not only uncovered the ATF-3/miR-590/GOLPH3 signaling pathway on regulating the breast cancer proliferation, but provides a new diagnosis marker and therapeutic target based on miRNA regulation for future breast cancer clinical treatment.

## Methods

### Cell culture

Human breast cancer cell lines MDA-MB-231 (human, ATCC® CRM-HTB-26™) and MCF-7 (human, ATCC® HTB-22™) were cultured in RPMI-1640 medium (Hyclone, Logan, UT) with 10% (*v*/v) fetal bovine serum (FBS) (Gibco, NY, USA), at 37°C, 5% CO2 atmosphere, supplemented with 100 U/ml penicillin and 100 μg/ml streptomycin sulfate (Sigma, USA).

### Plasmid and transfection

The human GOLPH3 expression vector was performed by cloning GOLPH3-coding cDNA sequence into vector. For knockdown of GOLPH3, two human Golph3 targeting shRNA sequences were cloned into plko.1 vector. The vectors construction was referenced from the previous study [8].

The 3′-UTR segment of wild-type GOLPH3 mRNA, which possessed the binding site for miR-590-3p, was amplified from MDA-MB-231 cells DNA and cloned into the luciferase reporter vector pGL3cM (Promega, Madison, WI, USA). The sequences of primers are as follows: PF: 5’-GGCGTCGACACAGTTCAGACAGAAGTCACAAAAA-3′ (Sal1 restriction enzyme); PR:5’-GGCTCTAGACACCATCTAGTACTTTTGCAATGAA-3′ (Xbal restriction enzyme). Using the QuickChange Lightning Multi Site-Directed Mutagenesis Kit (Agilent Technologies, Santa Clara, CA, USA), the mutant miRNA-binding sites were obtained by replacing the miRNA- binding site sequences.

ATF-3 siRNA sequence was referenced by the previous study [28].

### Luciferase assays

MDA-MB-231 cells at a density of 2.5 × 10^4^ cells/ well were seeded in 48-well plates. Cells were co-transfected with 150 ng of the UTR luciferase reporter, 5 ng Renilla vector, and 25 pmol miRNA-590-3p or control miRNAs (Biotend, Shanghai, China). After 24 h of transfection, the cells were harvested and lysed. The luciferase reporter activity was detected according to the instruction of the dual-Luciferase Assay system (Promega).

### Cell proliferation analysis

Cells were seeded in 96-well plates at an initial density of 2× 10^3^ cells/well and were used to perform the 3-(4,5-dimethylthiazol-2-yl)- 5-(3-carboxymethoxyphenyl) -2-(4-sul-fophenyl)-2H–tetrazolium (MTS) proliferation assay following the instruction of Cell Titer 96 AQueous One Solution Cell Proliferation Assay (Promega). The record absorbance at 490 nm was detected by microplate reader.

### Bromodeoxyuridine (BrdU) incorporation assay

Cells were seeded in 96-well plates at an initial density of 2× 103 cells/well with 10 μmol*/*L BrdU solution. After incubation for 2 h, PBS with 4% paraformaldehyde was used to fix the cells for 15 min. Washed cells with PBS and treated with DNase (Tiangen, China) for another 15 min at room temperature. Cells were washed with PBS again and stained with BrdU antibody (Abcam, Cambridge, Massachusetts, USA) for about 8 h at 4°C. Incubated the cells with secondary antibody at room temperature for 1 h. Then dyed cell nucleus with DAPI. Counted the BrdU-positive labeled cells in each group for at least 5 fields.

### Quantitative real- time PCR (qRT- PCR)

#### miRNA qRT-PCR

The total RNA was isolated using RNAiso plus (Takara, China).miRNA was subsequently reverse-transcribed to cDNA by using the miRNA-specific stem-loop reverse-transcription primer (Ribobio, China). The relative expression of each individual miRNAs was normalized to internal control U6. The qPCR reaction conditions were performed according to the instructions from Ribobio Co., Ltd.

#### mRNA qRT-PCR

cDNA was subsequently reverse-transcribed from total RNA by M-MLV Reverse Transcriptase (Takara). The PCR included 40 cycles of amplification using the Stratagene Mx3000P system with SYBR Green qPCR Mix (BioRad, Hercules, California). Expression of target genes (2^-ΔΔCt^) was normalized against GAPDH. Primer sequences are listed as follows: GAPDH (Gene ID: 2597) (forward: 5’-CTGGGCTACACTGAGCACC-3′; reverse: 5′- AAGTGGTCGT TGAGGGCAATG-3′), GOLPH3 (Gene ID:64,083) (forward: 5’-GCCTCCAGAAACGGTCCAG-3′; reverse: 5’-GTCAATACACCCTTTTCCACCA-3′), Cyclin E (Gene ID:898) (forward: 5’-CGCCTGCCGGGACTGGAG -3′; reverse: 5’-TCTTCCTGGAGCGAGCCG-3′), Cyclin D (Gene ID:595) (forward: 5’-GACCACCGAGG AGTTTAATCG-3′; reverse: 5’-GGGTGATCCCCTGATCCTTTG-3′), p21 (Gene ID:1026) (forward: 5’-TGTCCGTCAGAACCCATGC-3′; reverse: 5’-AAAGTCGAAGTTCCATCGCTC-3′).

#### Western blotting and antibodies

Cells were lysed with SDS buffer to obtain the protein which was used for further electrophoresis and transferred onto PVDF membranes (Millipore, Germany). Antibodies used in this study were listed as follows: anti-GAPDH (ab97626, Abcam, Cambridge, MA, USA), anti- GOLPH3 (ab91492, Abcam, Cambridge, MA, USA). The membrane was incubated with secondary antibodies. Signals were detected by enhanced chemiluminescence (ECL) western substrate (Thermo, Waltham, MA).

#### Flow cytometry

Cells were harvested and washed with PBS. After fixing by 70% alcohol over night, RNase A (20 mg/mL final concentration) was used. Then propidium iodide staining solution (50 mg/mL final concentration) were added to the cells and incubated for 15 min in the dark. Cells were analyzed by using a Cytomics FC 500 instrument (Beckman Coulter) equipped with CXP software.

#### mRNA level expression analysis in patients’ tissues samples

TCGA data we used in the study is publicly available, it can be accessed in the Broad GDAC Fire hose database (http://gdac.broadinstitute.org/). We can download data from the website. The Cancer Genome Atlas (TCGA) (http://cancergenome.nih.gov/). We used RSEM value for analysis to detect the difference of GOLPH3 expression between cancer samples and normal samples or at different stages. T-test was used for statistics.

#### Survival analysis

Whole mRNA expression data and clinical data of breast cancer samples were obtained from TCGA database. For survival analysis, patients were separated into two groups according to median expression level of GOLPH3.

#### Statistical analysis

Each experiment was performed at least 3 times (*n* ≥ 3). Statistical significance was determined between groups was determined by using the Student’s t-test or One way anova. Values were presented as the mean ± SD. *, **, *** means *P* < 0.05, *P* < 0.01, *P* < 0.001 respectively.

## Result

### Higher expression level of GOLPH3 in breast cancer is critically involved in the malignancy and poor prognostic

**Fig. 1:**
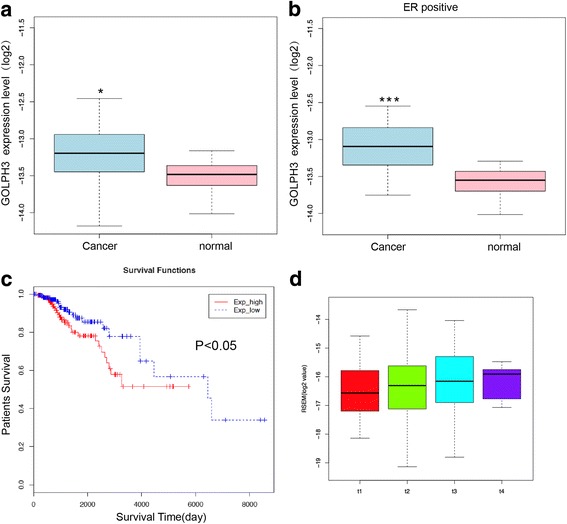
High expression level of GOLPH3 is critically involved in poor prognostic and malignancy. **a** GOLPH3 were upregulated in the breast cancer (light blue) compared with normal tissues (pink) in the Cancer Genome Atlas Database. *P < 0.05.**b** GOLPH3 level in ER positive samples, ***P < 0.001 (**c**) Kaplan–Meier (KM) survival curves of breast cancer patients are stratified by their expression level of GOLPH3. **d** GOLPH3 was associated with the T stage. Both survival information and genomic profiles provided by TCGA were used to determine whether the expression level show association with overall survival. The y-axis is survival probability of pateints. The x-axis is overall survival (days) of patients

We collected data from the TCGA database of cancer and normal samples to investigated whether breast cancer patients had aberrant circulating level of GOLPH3.We found that breast cancer patients (*n* = 1097) showed higher level of GOLPH3 compared with normal tissue (Fig. 1a). We also found the upregulation of GOLPH3 in ER positive (ER+) cancer tissues (*n* = 808) (Fig. 1b). Survival analysis revealed that survival rate of patients with higher GOLPH3 expression was dramatically lower than that of patients with low GOLPH3 expression (Fig. 1c). We found that the expression level of GOLPH3 was associated with the T stage in all the breast cancer samples (*n* = 1097), which suggested that upregulation of GOLPH3 was related with the increase of tumor (Fig. 1d).

### GOLPH3 promotes cell proliferation through regulating cell cycle

**Fig. 2:**
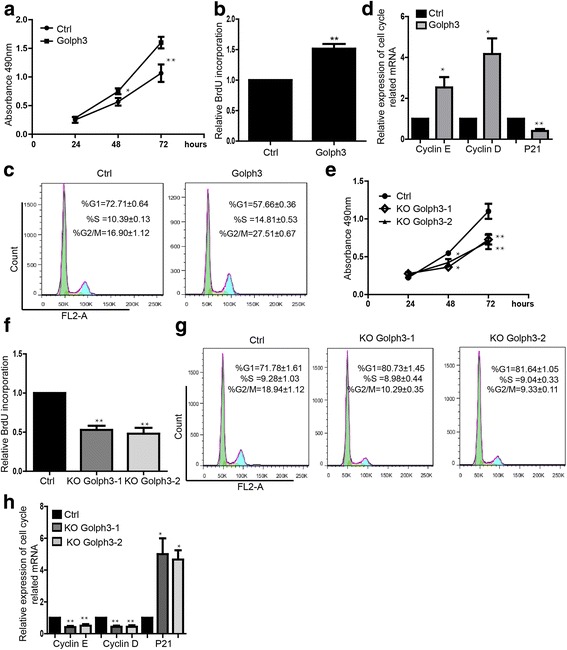
GOLPH3 regulates cell proliferation and cell cycle. **a** MTS assay showed promotion of cells proliferation by GOLPH3 overexpression during 24, 48, 72 h in MDA-MB-231. Data shown are means ± SD (*n* = 3). *P < 0.05, **P < 0.01. **b** BrdU incorporation assay showed the promotion of proliferation by overexpressing GOLPH3 in MDA-MB-231. Data shown are means ± SD (*n* = 3). **P < 0.01. **c** Flow cytometry assay showed the regulation of cell cycle by overexpressing GOLPH3. Data shown are means ± SD (*n* = 5). **d** Expression level of mRNA of cell cycle-related genes. Data shown are means ± SD (*n* = 3). *P < 0.05, **P < 0.01 versus the corresponding control. **e** Knockdown of GOLPH3 repressed cell proliferation during 24, 48, 72 h detected by MTS assay. Data shown are means ± SD (*n* = 3). *P < 0.05, **P < 0.01. **f** BrdU incorporation assay of cells with knockdown of GOLPH3. Data shown are means ± SD (*n* = 5). **P < 0.01.**g** Flow cytometry assay showed the cell cycle arrest at G1 phase by knockdown GOLPH3. Data shown are means ± SD (*n* = 5). **h** Expression level of mRNA of cell cycle-related genes. Data shown are means ± SD (*n* = 3). *P < 0.05, **P < 0.01 versus the corresponding control

We overexpressed the GOLPH3 and found that GOLPH3 promoted proliferation of breast cancer cells MDA-MB-231 (Fig. 2a) and MCF-7 (Additional file 1: Figure S1A) during 24, 48, 72 h. Significant higher BrdU incorporation showed the increase of DNA replication and cell division after overexpressing GOLPH3, which showed the promotion of breast cancer cell proliferation (Fig. 2b, Additional file 1: Figure S1B). We further found that there was significant decrease in the proportion of cells in G1 phase and an increase in the proportion of cells in S, and G2/ M phases in GOLPH3 overexpression group (Fig. 2c).Cell cycle relative genes Cyclin E, Cyclin D were upregulated and cyclin-dependent kinase (CDK) inhibitor P21 [8] was downregulated by overexpressing GOLPH3 (Fig. 2d).

On the contrary, we also downregulated the expression of GOLPH3 and found that proliferation of MDA-MB-231 cells were repressed in the GOLPH3 knockdown group (Fig. 2e). This result was also confirmed by BrdU assay as well (Fig. 1f). Additionally, the proportion of cells in G1 phase was increased and decreased in the S, G2/M phase by knockdown of the GOLPH3 (Fig. 1g).We also found that cyclin D, cyclin E were downregulated and the P21 were upregulated in the GOLPH3 knockdown group (Fig. 2h).

### miR-590-3p represses breast cancer cells proliferation and directly targeted GOLPH3

**Fig. 3:**
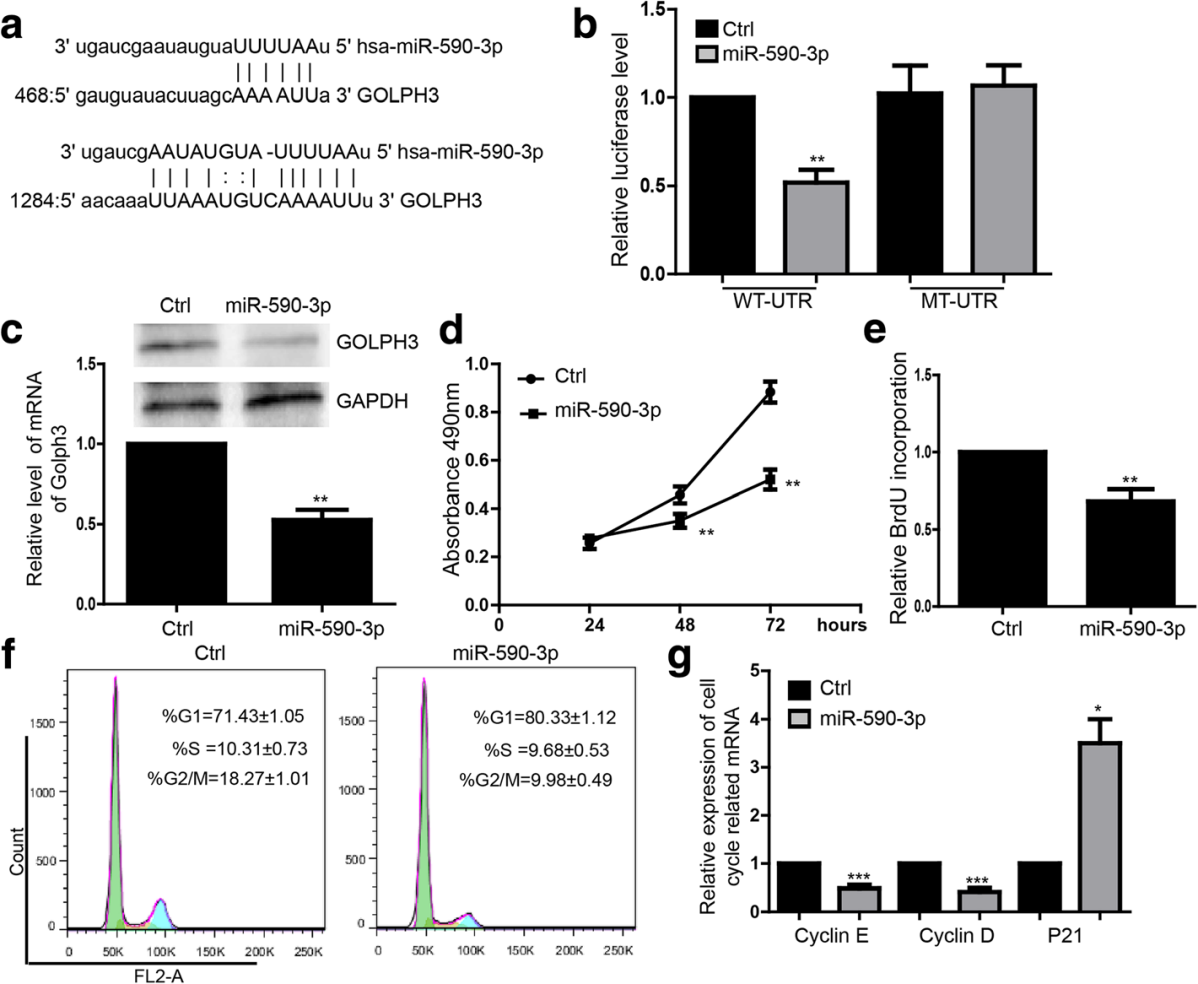
miR-590-3p represses breast cancer cells proliferation and directly targets GOLPH3. **a** The GOLPH3 3′UTR reporter in target validation of miR-590-3p. **b** Luciferase analysis showed the inhibitory effect of miR-590-3p on expression of the luciferase reporter gene by binding GOLPH3 3’UTR. **P < 0.01. **c** q-PCR and western blot showed the down-regulated GOLPH3 protein in MDA-MB-231 by overexpressing miR-590-3p. Data shown are means ± SD (*n* = 3). **P < 0.01. **d** MTS assay showed miR-590-3p repressed cell proliferation during 24, 48, 72 h. Data shown are means ± SD (*n* = 3).**P < 0.01. **e** BrdU incorporation assay. Data shown are means ± SD (*n* = 5). **P < 0.01. **f** Flow cytometry assay showed the cell cycle arrest at G1 phase by overexpressing miR-590-3p. Data shown are means ± SD (*n* = 5). **g** Expression level of mRNA of cell cycle-related genes. Data shown are means ± SD (*n* = 3). *P < 0.05, ***P < 0.005 versus the corresponding control

By using online bioinformatics assay (Miranda, targetscan), we found the miRNA-targeting sites of miR590-3p on the 3′-UTR of GOLPH3 (Fig. 3a). The overexpression of pre-miR-590-3p significantly reduced the luciferase activities of the wild type GOLPH3 3 ‘UTR reporter compared with the negative control. In contrast, when mutant sequence happened at the binding sites, the luciferase level of mutant UTR group showed no significant difference from control group (Fig. 3b). To validate the influence of the miR-590-3p on GOLPH3 expression, we further determined that overexpression of miR-590-3p downregulated the expression of GOLPH3 on both mRNA and protein level (Fig. 3c).

Further, we upregulated miR-590-3p and studied the effect on the proliferation. MTS assay showed that the proliferation was inhibited during 24, 48 and 72 h (Fig. 3d), and the result was also confirmed by BrdU incorporation assay (Fig. 3e). Additionally, there was significantly increased of cell population respectively at phase G1and decreased at phase S, G2/M (Fig. 3f). The mRNA expression levels of cyclin E and D were found to be downregulated and the p21 was upregulated in miR-590-3p overexpression MDA-MB-231 cells (Fig. 3g).

### miR-590-3p/GOLPH3 signaling regulates the proliferation of breast cancer cells

**Fig. 4:**
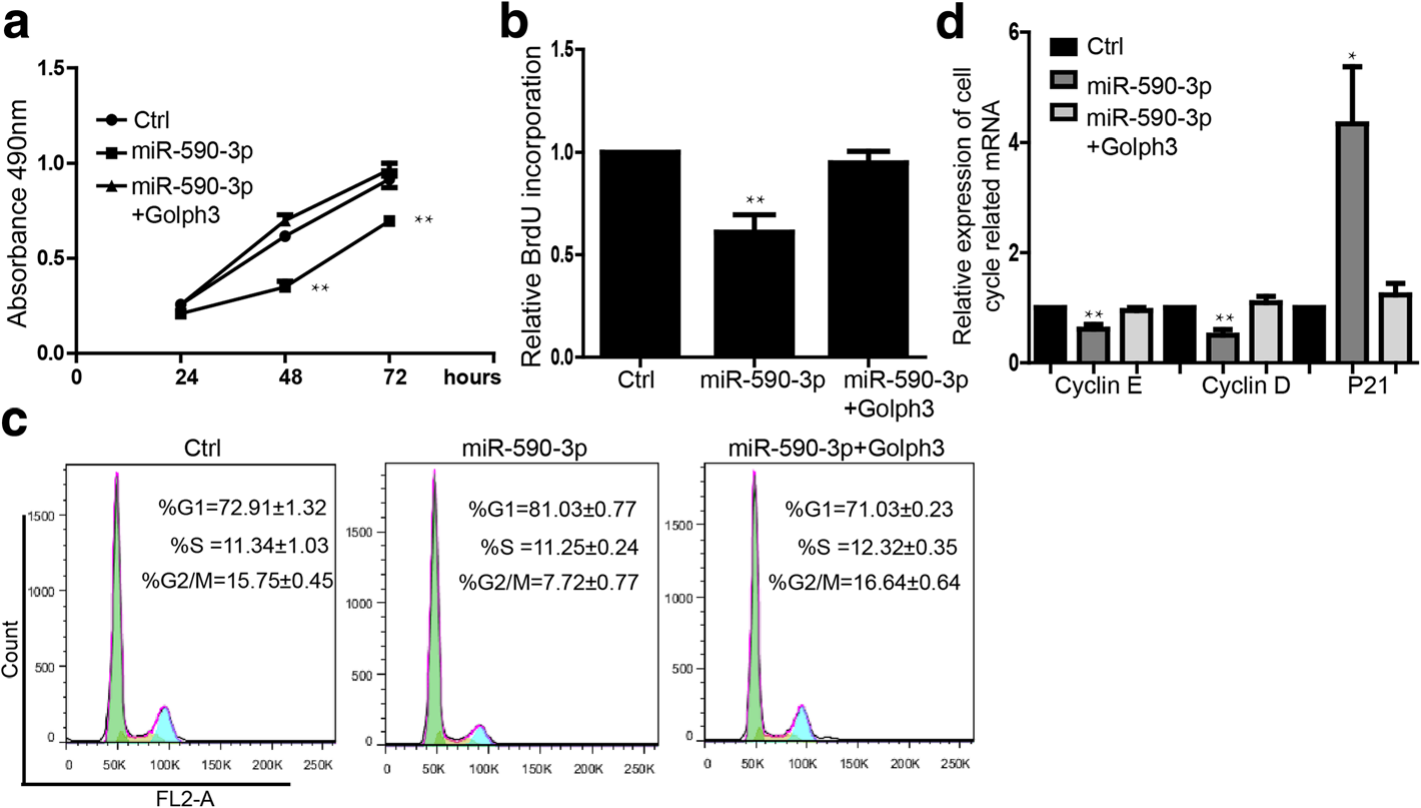
miR-590-3p repressed the proliferation by targeting GOLPH3. **a** MTS proliferation assay showed overexpression of GOLPH3 could rescue miR-590-3p repressing cell proliferation during 24, 48, 72 h. Data shown are means ± SD (n = 3), **P < 0.01. **b** BrdU incorporation assay in rescue experiment. Data shown are means ± SD (*n* = 3), **P < 0.01. **c** Flow cytometry assay in rescue experiment. Data shown are means ± SD (*n* = 5). **d** Expression level of mRNA of cell cycle-related genes. Data shown are means ± SD (*n* = 3). **P < 0.01 versus the corresponding control

In order to determine whether the function of miR-590-3p is directly mediated by the inhibition of GOLPH3 expression, we performed the rescue experiment. MTS assay showed that the proliferation inhibition caused by miR-590-3p could be restored by overexpression of GOLPH3 (Fig. 4a). BrdU incorporation assay also confirmed it (Fig. 4b). Additionally, overexpression of GOLPH3 was able to rescue the influence of cell cycle induced by miR-590-3p (Fig. 4c). Expression of miR-590-3p modulated cell cycle associated genes on mRNA level, while these regulation overexpressing GOLPH3 (Fig. 4d).

### Downregulation of ATF-3 suppresses the proliferation of breast cancer cells

**Fig. 5:**
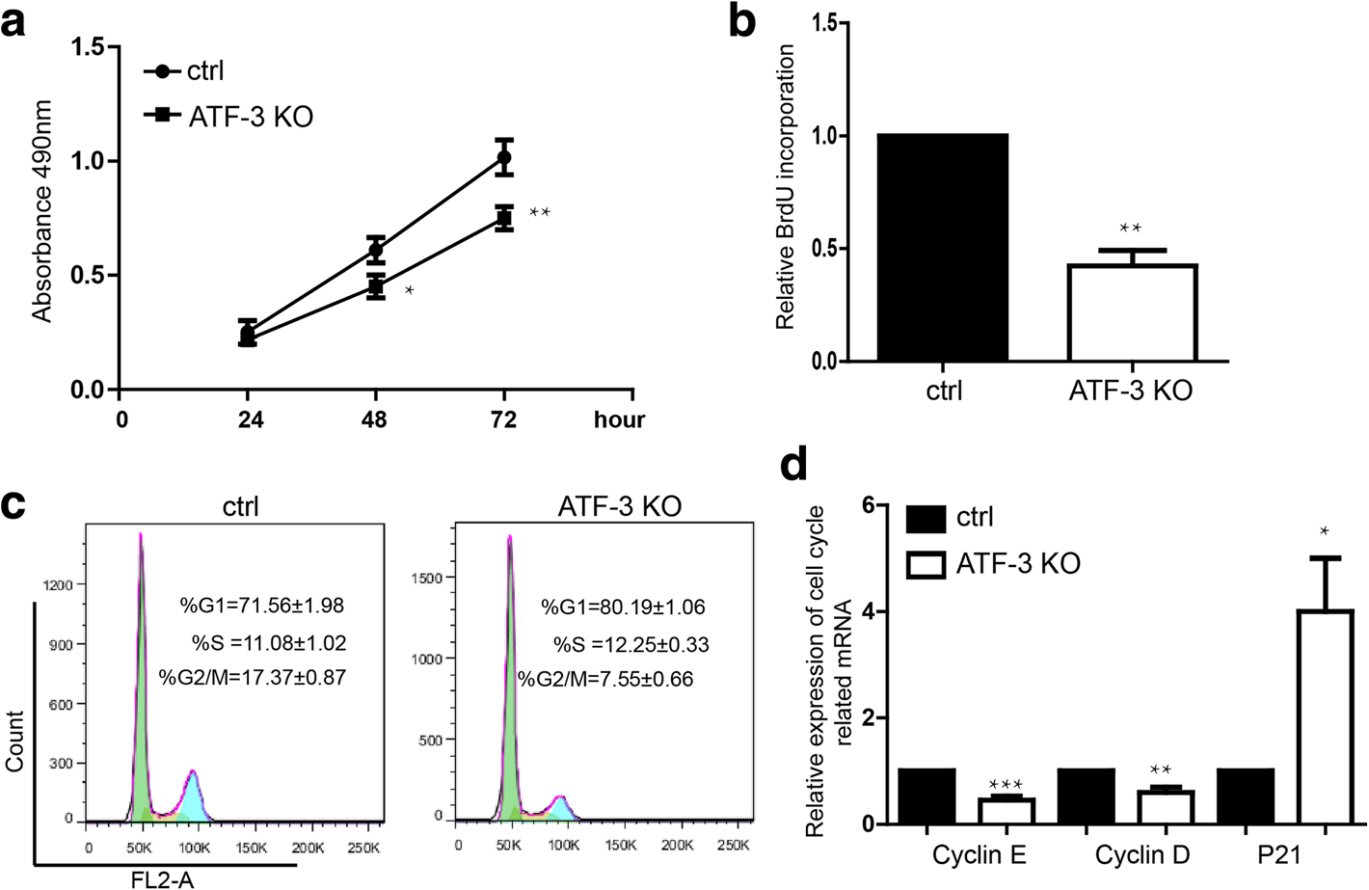
Knockdown of ATF-3 inhibits the proliferation of breast cancer cells. **a** MTS proliferation assay showed downregulation of ATF-3 could inhibit proliferation during 24, 48, 72 h in MDA-MB-231. Data shown are means ± SD (*n* = 5), *P < 0.05. **b** BrdU incorporation assay. Data shown are means ± SD (*n* = 3), **P < 0.01. **c** Flow cytometry assay showed the increase of cell proportion in G1 phase of the ATF-3 knockdown group. Data shown are means ± SD (*n* = 5). **d** Detection of expression cell cycle-related genes by qPCR. Data shown are means ± SD (*n* = 3). *P < 0.05, **P < 0.01, ***P < 0.001 versus the corresponding control

Previous study indicated that the transcription of miR-590 could be inhibited by ATF-3 [27].Then we detected the function of ATF-3 on regulating the proliferation of breast cancer cells. We found that downregulation of ATF-3 by transfecting the siRNA [28] inhibited the proliferation detected by MTS assay (Fig. 5a, Additional file 2: Figure S2A) and BrdU incorporation assay (Fig. 5b). We further determined that downregualtion of ATF-3 also induced more cells arrest at G1 phase (Fig. 5c). Knockdown of ATF-3 repressed the expression of Cyclin E and Cyclin D and upregulated the P21 (Fig. 5d).

### ATF-3 modulates miR-590-3p/ GOLPH3 signaling to repress the proliferation of breast cancer cells

**Fig. 6:**
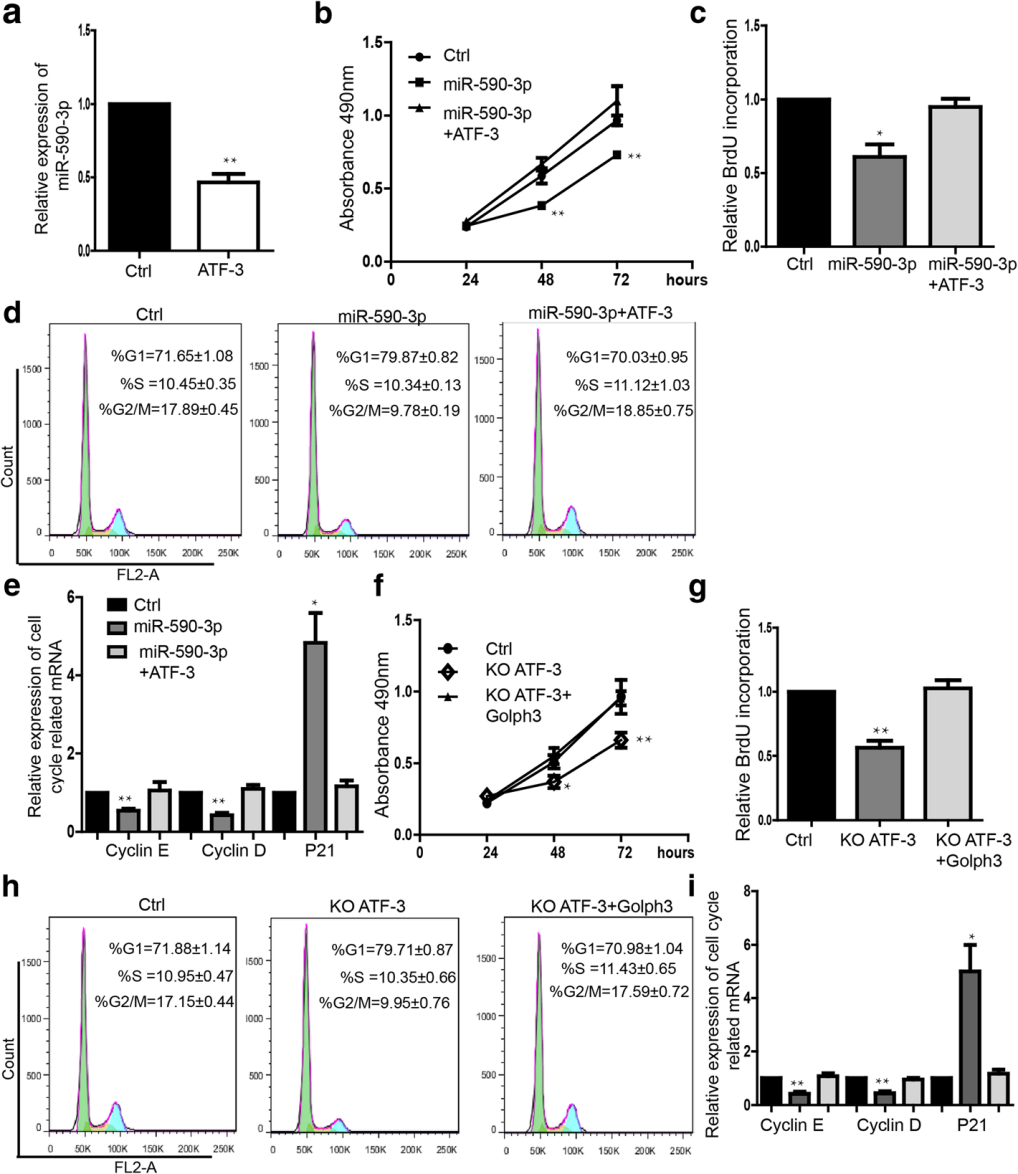
Through regulating miR-590-3p/GOLPH3 signaling pathway, can ATF-3 represses proliferation of breast cancer cells. **a** Ectopics expression of ATF-3 inhibited the expression of miR-590-3p. Data shown are means ± SD (*n* = 5), **P < 0.01 (**b**) MTS proliferation assay showed overexpression of ATF-3 could rescue miR-590-3p repressing cell proliferation during 24, 48, 72 h in MDA-MB-231. Data shown are means ± SD (*n* = 3), **P < 0.01. **c** BrdU incorporation assay. Data shown are means ± SD (*n* = 3), *P < 0.05. **d** Flow cytometry assay showed overexpression ATF-3 rescued the G1 phase arrest caused by miR-590-3p. Data shown are means ± SD (*n* = 5).**e** Expression level of mRNA of cell cycle-related genes. Data shown are means ± SD (*n* = 3). **f** MTS proliferation assay showed that knockdown of ATF-3 repressed cell proliferation, which could be rescued by overexpressing GOLPH3 during 24, 48, 72 h in MDA-MB-231. Data shown are means ± SD (*n* = 3), *P < 0.05, **P < 0.01. **g** BrdU incorporation assay. Data shown are means ± SD (n = 3), **P < 0.01. **h** Flow cytometry assay showed overexpression GOLPH3 rescued the influence of cell cycle caused by knockdown of ATF-3. Data shown are means ± SD (*n* = 5). **i** Expression level of mRNA of cell cycle-related genes. Data shown are means ± SD (*n* = 3). *P < 0.05, **P < 0.01 versus the corresponding control

We also found that overexpression of ATF-3 repressed the expression of miR-590-3p (Fig. 6a).The proliferation ability could be restored by overexpressing ATF-3 in miR-590 overexpressed MDA-MB-231 cells (Fig. 6b and c) and in MCF-7 (Additional file 3: Figure S3A). Additionally, overexpression of ATF-3 blocked the influence of cell cycle induced by overexpression of miR-590-3p (Fig. 6d). The expression level of Cyclin E, D and P21 regulated by miR-590-3p could also be rescued to be similar with the level in control group by ATF-3 overexpressing (Fig. 6e).Further we found that downregulation of ATF-3 caused proliferation inhibition which could be restored by overexpressing GOLPH3 in both MDA-MB-231 and MCF-7 cells (Fig. 6f and  g) and MCF-7 (Additional file 3: Figure S3B). The arrest of phase G1 induced by knockdown of ATF-3 was rescued by upregulation of GOLPH3 (Fig. 6h). Similarly, ATF-3 knockdown induced the significant downregulation of Cyclin E, Cyclin D and upregulation of P21, which could be blocked by overexpressing GOLPH3 as well (Fig. 6i).

## Discussion

In this study, we demonstrated the regulatory function of ATF-3/miR-590-3p/GOLPH3 signaling pathway in breast cancer on regulating the proliferation of breast cancer cells. Multiple cellular pathways were dysregulated in breast cancer [29]. Approximately 5% of breast cancer patients were diagnosed with metastatic disease at initial presentation [30]. Among the entire cellular pathway, proliferation is a process which is essential to cancer cell viability and invasion as well. Recently, a growing number of reports recognize novel therapeutic targets, including of the proteins in control of Golgi homeostasis. Golgi associated proteins can regulate tumorigenesis from different aspects, such as cell apoptosis [31], invasion and motility [32]. Upregulation of GOLPH3 promotes proliferation of prostate cancer [33]. Knockdown of the GOLPH3 prevented the proliferation in human rhabdomyosarcoma cell [34]. Here we found that overexpression of GOLPH3 promoted the breast cell proliferation with the increase of cell proportion of S and G2/M phases, which was also deteremined by previous study [13]. In the contrary, downregulation of GOLPH3 suppressed the proliferation. The critical golgi related gens, GOLPH2, GOLM1 and GP73, can also be the potential biomarkers in prostate cancer and liver cancer [35, 36].GOLPH3 was also reported to be the potential biomarker in some kinds of cancers. Upregulation of GOLPH3 correlates with poor response to prognosis in locally advanced rectal cancer [37] and NSCLC [12].We found that GOLPH3 was significantly upregulated in breast cancer tissues compared with the normal tissues. Higher expression of GOLPH3 was related to the poor prognosis. These results showed that the GOLPH3 expression not only regulates the proliferation of breast cancer cells, but suggested that the expression of GOLPH3 might the potential biomarker of early diagnosis of breast cancer in future clinical treatment.

miRNAs have been reported to be the biomarkers of cancer detection and have directly or indirectly relation with tumorigenesis and further development [38]. Previous study showed that miR-126, a tumor suppressor, promotes apoptosis or induced proliferation repression in many kinds of caners [39–41] and also was reported to target GOLPH3 to suppress proliferation, migration and invasion in esophageal squamous cell carcinoma [20]. In gastric cancer cells miR-134 suppresses cell proliferation via targeting of GOLPH3 [42]. miR-590 was found to be upregulated in many different kinds of cancers to modulate proliferation and invasion of tumor cells [43, 44].We found that miR-590-3p significantly inhibited the breast cancer cell proliferation and influenced the cell cycle and the related genes expression. Additionally, we found that miR-590-3p directly targets GOLPH3 3’UTR to downregulate the expression. Overexpression of GOLPH3 blocked the suppression of miR-590-3p on proliferation. These results uncovered the miR-590-3p/GOLPH3 signaling regulating the proliferation of breast cancer cells and suggested the novel targets for the anti-breast cancer agents development. Further we will detect the molecular pathway of GOLPH3 to investigate the downstream critical direct genes which regulate the cell cycle.

miRNA expression is regulated by upstream regulators. Previous study has showed that there is a negative feedback regulation of miR-590 and ATF-3 in breast cancer. Inhibition of ATF-3 significantly upregulated the expression of miR-590 [27]. In this study we found that overexpression of ATF-3 blocked the repression of inhibition caused by miR-590. Moreover, overexpression of GOLPH3 restored the proliferation inhibited by ATF-3 knockdown. These results indicated that ATF-3 modulate miR-590-3p/ GOLPH3 signaling pathway to repress the proliferation of breast cancer cells. This result showed the new downstream target signaling pathway of ATF-3 and suggested the critical regulatory relationship between ATF-3 and golgi related genes.

Previous study showed that overexpression of Gologh3 in 7 kinds of breast cancers promoted the proliferation with the downregulation of cyclin-dependent kinase (CDK) inhibitor p21Cip1 and upregulated the CDK regulator cyclin D1 [8]. Our study also showed that ATF-3/miR-590-3p/GOLPH3 pathway regulates the proliferation of MCF7 and MDA-MB-231 cells which represent two distinct genetic backgrounds. This result indicated that ATF-3/miR-590-3p/GOLPH3 could be the effective targets for future clinical diagnosis and treatment. Further we will detect whether there are unique pathway related to Gologh3 in different kinds of breast cancer cells.

In summary, our results demonstrated the ATF-3/ miR-590-3p/GOLPH3 signaling pathway on regulating the proliferation and the cell cycle of breast cancer cells. Uncovered the regulation mechanism of ATF-3 on golgi related genes by miRNA. Our data provided new insights into the potential biomarkers and mechanisms of breast cancer oncogenesis and also suggest a potential application of treatment in the future.

## Conclusions

1. GOLPH3 is upregulated in breast cancer and was related to the poor prognosis.
2. miR-590 targets GOLPH3 to inhibit the breast cancer cell proliferation.
3. ATF-3 repressed miR-590-3p expression to regulate miR-590/GOLPH3 pathway, modulating proliferation of breast cancer cells.
4. ATF-3/miR-590/GOLPH3 signaling pathway can be a novel therapeutic target and diagnosis markers in future treatment.

## Additional files

Additional file 1: Figure S1. Related to Fig. 2. Golph3 regulates cell proliferation of MCF-7 cells (A) MTS assay showed promotion of cells proliferation because of Golph3 overexpression during 24, 48, 72 h in MCF-7 cells . Data shown are means ± SD (*n* = 3). **P < 0.01. (B) BrdU incorporation assay . Data shown are means ± SD (*n* = 3). *P < 0.05. (TIFF 3999 kb)

Additional file 2: Figure S2. Related to Fig. 5. knockdown of ATF-3 inhibited proliferation of MCF-7 cells. (A) MTS assay showed downregulation of ATF-3 by transfecting siRNA inhibits proliferation during 24, 48, 72 h in MCF-7 cells . Data shown are means ± SD (*n* = 4). *P < 0.05. (TIFF 96 kb)

Additional file 3: Figure S3. Related to Fig. 6. ATF-3/miR-590-3p/Golph3 regulates the proliferation of MCF-7 cells (A) MTS proliferation assay showed overexpression of ATF-3 could rescue miR-590-3p repressing cell proliferation during 24, 48, 72 h in MCF-7. Data shown are means ± SD (*n* = 3), **P < 0.01. (B) MTS proliferation assay showed knockdown of ATF-3 repressed cell proliferation, which could be rescued by overexpress Golph3 during 24, 48, 72 h in MCF-7. Data shown are means ± SD (*n* = 3), **P < 0.01. (TIFF 223 kb)

## Abbreviations

ATF3: Activating transcription factor 3
BrdU: Bromodeoxyuridine
CDK: Cyclin-dependent kinase
GOLPH3: Golgi phosphoprotein 3
miRNAs: MicroRNAs
MTS: 3-(4,5-dimethylthiazol-2-yl)-5-(3-carboxymethoxyphenyl) -2-(4-sul-fophenyl)-2H–tetrazolium
NSCLC: Non-small-cell lung cancer
qRT- PCR: Quantitative real- time PCR
TCGA: The Cancer Genome Atlas
UTR: Untranslated region
