# Supplementary figures and images for: ATF-3/miR-590/GOLPH3 signaling pathway regulates proliferation of breast cancer
Source: BMC Cancer. 2018 Mar 9;18:255. doi: 10.1186/s12885-018-4031-4 (PMC6389151; doi:10.1186/s12885-018-4031-4)

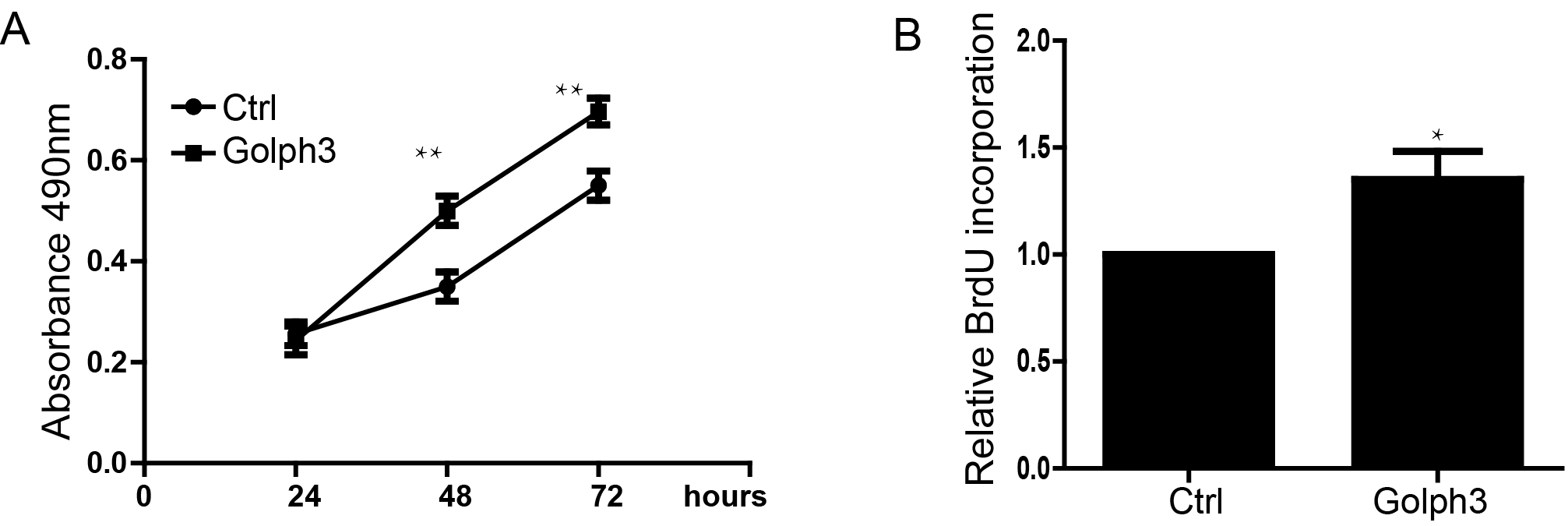

Supplement: Supplementary file 1 — Related to Fig. 2. Golph3 regulates cell proliferation of MCF-7 cells (A) MTS assay showed promotion of cells proliferation because of Golph3 overexpression during 24, 48, 72 h in MCF-7 cells . Data shown are means ± SD (n = 3). **P < 0.01. (B) BrdU incorporation assay . Data shown are means ± SD (n = 3). *P < 0.05. (TIFF 3999 kb) [file 12885_2018_4031_MOESM1_ESM.tif]

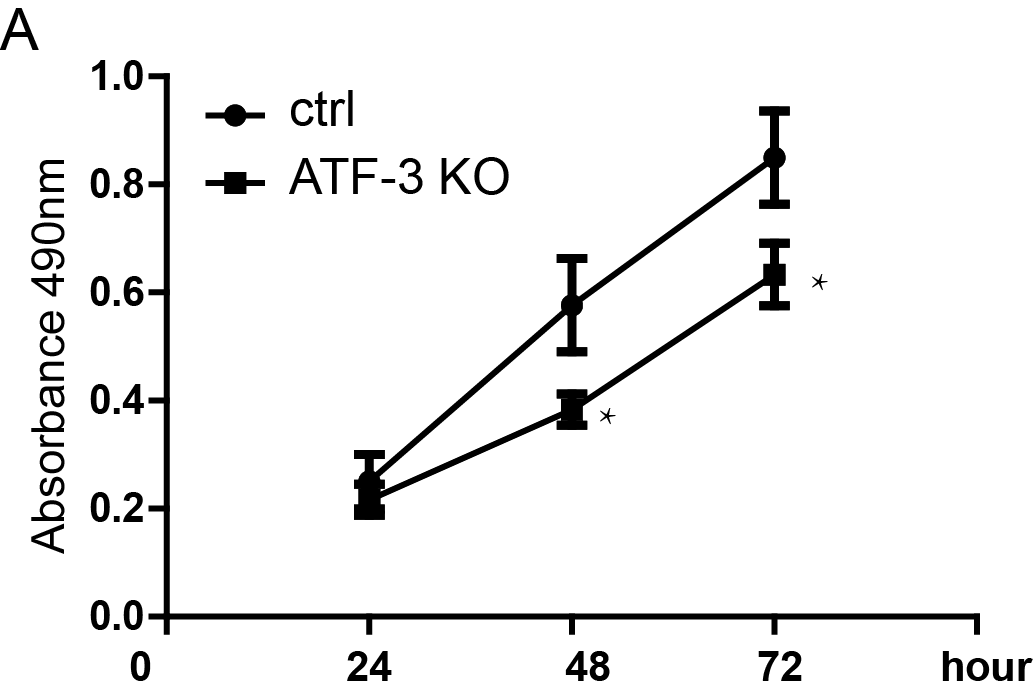

Supplement: Supplementary file 2 — Related to Fig. 5. knockdown of ATF-3 inhibited proliferation of MCF-7 cells. (A) MTS assay showed downregulation of ATF-3 by transfecting siRNA inhibits proliferation during 24, 48, 72 h in MCF-7 cells . Data shown are means ± SD (n = 4). *P < 0.05. (TIFF 96 kb) [file 12885_2018_4031_MOESM2_ESM.tif]

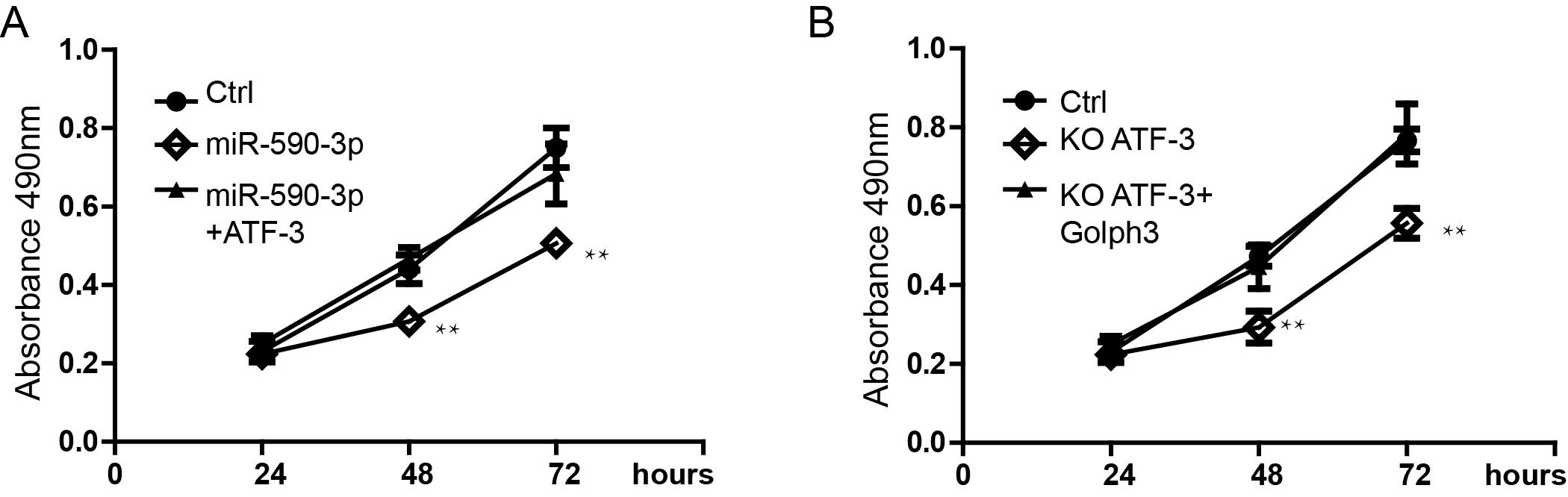

Supplement: Supplementary file 3 — Related to Fig. 6. ATF-3/miR-590-3p/Golph3 regulates the proliferation of MCF-7 cells (A) MTS proliferation assay showed overexpression of ATF-3 could rescue miR-590-3p repressing cell proliferation during 24, 48, 72 h in MCF-7. Data shown are means ± SD (n = 3), **P < 0.01. (B) MTS proliferation assay showed knockdown of ATF-3 repressed cell proliferation, which could be rescued by overexpress Golph3 during 24, 48, 72 h in MCF-7. Data shown are means ± SD (n = 3), **P < 0.01. (TIFF 223 kb) [file 12885_2018_4031_MOESM3_ESM.tif]
